# Potential effect of the non-neuronal cardiac cholinergic system on hepatic glucose and energy metabolism

**DOI:** 10.3389/fcvm.2024.1381721

**Published:** 2024-05-16

**Authors:** Atsushi Kurabayashi, Waka Iwashita, Kaoru Furihata, Hideo Fukuhara, Keiji Inoue

**Affiliations:** ^1^ Department of Pathology, Kochi Medical School, Nankoku, Japan; ^2^ Department of Urology, Kochi Medical School, Nankoku, Japan

**Keywords:** non-neuronal cardiac cholinergic system, remote ischemic preconditioning, acetylcholine, vagus nerve, parasympathetic nervous system, central nervous system, liver, glucose metabolism

## Abstract

The vagus nerve belongs to the parasympathetic nervous system, which is involved in the regulation of organs throughout the body. Since the discovery of the non-neuronal cardiac cholinergic system (NNCCS), several studies have provided evidence for the positive role of acetylcholine (ACh) released from cardiomyocytes against cardiovascular diseases, such as sympathetic hyperreactivity-induced cardiac remodeling and dysfunction as well as myocardial infarction. Non-neuronal ACh released from cardiomyocytes is believed to regulate key physiological functions of the heart, such as attenuating heart rate, offsetting hypertrophic signals, maintaining action potential propagation, and modulating cardiac energy metabolism through the muscarinic ACh receptor in an auto/paracrine manner. Moreover, the NNCCS may also affect peripheral remote organs (e.g., liver) through the vagus nerve. Remote ischemic preconditioning (RIPC) and NNCCS activate the central nervous system and afferent vagus nerve. RIPC affects hepatic glucose and energy metabolism through the central nervous system and vagus nerve. In this review, we discuss the mechanisms and potential factors responsible for NNCCS in glucose and energy metabolism in the liver.

## 1 Introduction

Recent studies on the regulation of the cholinergic system in the cardiovascular field have provided evidence for its significant contribution. Acetylcholine (ACh) is a neurotransmitter in the autonomic nervous system, which consists, in part, of the sympathetic nervous system. Vagus nerve stimulation (VNS) has shown promising protective effects against heart failure in humans, which is consistent with the results of animal studies (1, 2).

The nerve ends of the parasympathetic nervous system (PNS) in the heart are located mainly in the sinus and atrioventricular nodes, with only a sparse distribution in the ventricles, whereas the nerve ends of the sympathetic nervous system (SNS) are distributed throughout the ventricles (3–7). Focusing on this difference, Kakinuma et al. suggested that a novel system within the heart is responsible for ACh synthesis independent of the PNS (8). It is non-neuronal ACh synthesis in the heart, or non-neuronal cardiac cholinergic system (NNCCS)—ACh production by cardiomyocytes. Saw et al. examined the molecular mechanism underlying the protective effect of NNCCS mediated by ACh-initiated muscarinic ACh receptor signaling (9). NNCCS activates the phosphatidylinositol-3-kinase (PI3 K)/protein kinase B (Akt) signaling pathway and increases hypoxia-inducible factor (HIF)-1α expression under normoxic conditions. Increased PI3 K and Akt phosphorylation prevents the binding of von Hippel-Lindau protein (pVHL) to HIF-1α. HIF-1*α* and HIF-1β dimerize to form master transcription factors that induce transcription of various hypoxia-related genes, ultimately leading to angiogenesis (e.g., VEGF), glycolysis [e.g., glucose transporter (GLUT) 4] and minimize the death of myocardial cells due to ischemia (9). Furthermore, Kakinuma et al. found that the release of VNS and ACh activates a self-defense mechanism regulated by HIF-1 (10). They demonstrated that NNCCS augmentation in a transgenic mouse, which was a representative useful model of activated NNCCS alone, overexpressing cardiomyocyte-specific choline acetyltransferase (ChAT-tgm), accelerated ischemia and hypoxia-resistant potency on the heart (11). They suggested that NNCCS plays an important role in cardia-homeostasis (i.e., cell–cell communication, angiogenesis, oxygen consumption, anti-ischemia, and resistance to hypoxia) (8, 11–13).

It was suggested that brief episodes of ischemia in one vascular bed protected remote, virgin myocardium from sustained coronary artery occlusion (14), and then, the method of remote ischemic preconditioning (RIPC) using a tourniquet was introduced (15). Donato et al. suggested that cardioprotection resulting from RIPC [i.e., hindlimb ischemia–reperfusion (IR)] involved an afferent neural signal because protection was lost after the spinal cord was sectioned (16). In addition, activation of the efferent PNS and muscarinic cholinergic receptors was lost after vagotomy and following atropine administration. As efferent neural pathway of RIPC, they reported that electrical stimulation of the vagal nerve could induce cardioprotection mimicking RIPC. ACh increases the expression of HIF-1α and GLUT 4 (11). In this way, the action mechanism of RIPC, modulated by cholinergic stimuli, is similar to NNCCS. Possibility to activate NNCCS is thought about so that IR activates RIPC. ACh participates in the functional regulation of various organs. The hypothesis that activation of the parasympathetic system by NNCCS may have an influence on other organs not only the heart is suggested.

We demonstrated that conditional *VHL* gene deletion results in insulin-independent hypoglycemia through the upregulation of hepatic insulin-like growth factor I receptor (IGF-IR), HIF-1α, and GLUT1 (17). Furthermore, Oikawa et al. reported that ChAT-expressing HEK293 cells (i.e., cells with an activated NNCCS) exhibited increased IGF-IR, HIF-1α, and GLUT1 protein expression as well as enhanced glucose uptake (18). It is known that PNS participates in the glucose metabolism in the liver (19). Based on these similarities with a focus on ACh, HIF-1α and GLUT, we discuss whether NNCCS participates in glucose and energy metabolism in the liver and delineate its mechanism.

## 2 Association of RIPC, NNCCS, and CNS

The vagus nerve and ACh play an important role in communicating with the brain and organs (e.g., heart and liver) via RIPC (16, 20, 21). Cardioprotection induced by RIPC involves afferent neural signals and efferent parasympathetic nerve signals (16). In addition, hindlimb IR activated the central nervous system (CNS), including solitary tract (NTS) and the dorsal motor vagal nucleus (DMX). Following the discovery of NNCCS, evidence of the significant role of this system in the heart has accumulated. At first, non-neuronal ACh released from cardiomyocytes is believed to regulate key physiological cardiac functions (e.g., heart rate, offsetting hypertrophic signals, maintaining action potential propagation, and modulating cardiac energy metabolism) (9). In addition, Kakinuna et al. demonstrated that RIPC through hindlimb IR upregulated cardiac ACh synthesis through NNCCS (12, 22). Moreover, ChAT-tgm exhibited a greater activation signal (i.e., c-fos-positive) in the neuronal cells around the NTS in the medulla oblongata and exhibited ascending vagus nerve effects similar to vagus nerve stimulation (23). Thus, NNCCS may activate CNS (i.e., the center of the PNS) and affect to the peripheral organs (e.g., liver).

## 3 NNCCS in the liver

The vagus nerve has important functions in modulating inflammation (24). The vagus nerve acts through the α7 nicotinic ACh receptor (α7nAChR) expressed on the surface of Kupffer cells to inhibit the production of the pro-inflammatory cytokines tumor necrosis factor (TNF) and interleukin (IL)-6 (25, 26). Insulin action on the insulin receptors in the central nervous system activates hepatic IL-6 signaling by suppressing vagal activity (26). Although the possibility of the non-neuronal cholinergic system in rat hepatocytes was discussed immunohistochemically (e.g., ChAT) (27), the clear evidence that hepatocytes produce ACh is not provided. Oikawa et al. revealed that the hepatic expression of TNF-α, IL-6, and IL-1β mRNA were all significantly decreased in ChAT-tgm compared with wild-type (WT) mice (control) (28). Kupffer cells are considered the primary source of cytokines; therefore, using cultured Kupffer cells, Oikawa et al. assessed these factors. Interestingly, the expression of TNF-α, IL-6, and IL-1β mRNA was also reduced in Kupffer cells derived from ChAT-tgm compared with those derived from WT mice (28). Vagotomy reversed this decrease in these interleukins in ChAT-tgm-derived Kupffer cells.

In murine nonalcoholic steatohepatitis (NASH) model, both hepatic vagotomy and α7nAChR knockout upregulated pro-inflammatory cytokine (e.g., TNF-α, IL-12). Moreover, α7nAChR agonist suppressed these cytokines expression of primary Kupffer cells cultured with palmitic acid, lipotoxic free fatty acid, and lipopolysaccharide (LPS) similar to NASH condition (29). In addition, the serum levels of TNF-α and IL-6 by LPS administration were both attenuated in ChAT-tgm (28). In *in vivo* rat liver, vagotomy reduces the production of anti- inflammatory hormone and cytokine (i.e., corticosterone and IL-10) and increases LPS-stimulated TNF synthesis, whereas electric vagus nerve stimulation decreased TNF synthesis without increasing corticosterone and IL-10 (24). Increasing of cytokine production by Kupffer cells under vagotomy may derive from these humoral mechanisms differ from mechanisms of vagus nerve stimulation (24). Moreover, the spleen is an important source of TNF-α production under septic condition (30). Thus, although vagus nerve stimulation by NNCCS and/or RIPC may suppress the inflammatory response of Kupffer cells under inflammatory conditions, further studies with NNCCS are needed.

In addition, autonomic nerve-mediated crosstalk between CNS and peripheral organs functions in the maintenance of glucose/energy metabolism and homeostasis. Thus, we hypothesized that NNCCS may affect on glucose metabolism in the liver and moreover, disorder of carbohydrate metabolism.

## 4 NNCCS and RIPC in diabetes mellitus (DM)

DM is a frequent comorbidity associated with cardiovascular disease and the occurrence of myocardial infarction (31). It is considered an independent risk factor for the development of myocardial infarction and ischemic heart disease. Patients with DM are also more likely to suffer from major perioperative adverse cardiac events compared with the non-diabetic population (32). RIPC has some similarities with exercise and may be induced by brief periods of ischemia and reperfusion of a limb. It can also be performed in individuals who cannot exercise. Using streptozotocin (STZ)-induced type 1 DM mice (T1DMm), Munasinghe et al. revealed that under T1DM conditions, dysregulation of the NNCCS (i.e., DM induced downregulation of ChAT in the heart) and reduced ACh levels were associated with impaired cardiac function [i.e., increased left ventricular (LV) end-diastolic and -systolic volume and reduced ejection fraction and LV end-systolic pressure] (33). Increasing ACh levels in the heart of ChAT-tgm could prevent these effects. Using a db/db mouse (db/db-m) as a type 2 diabetic model and db/db-ChAT-tgm, Saw et al. demonstrated that although GLUT4 was significantly decreased in the heart of db/db mice in a progressive stage of DM, in db/db-ChAT-tgm, the expression of type 2 muscarinic ACh receptors and its downstream targets, HIF-1α and GLUT4, in the heart were increased compared with those in db/db-m (34). These results suggest that the activation of cardiac ACh synthesis through NNCCS by RIPC (i.e., hindlimb IR) upregulated GLUT4 expression, suggesting increased glucose uptake under type 2 DM conditions. IR induced by three minutes of ischemia repeated three times increases ChAT expression as well as ACh and adenosine triphosphate (ATP) levels in the heart (22). As mentioned earlier, RIPC and/or NNCCS is thought to act on the liver, and it is possible that the changes in glucose metabolism (i.e., increasing glucose uptake) in the heart mentioned in this section also occur in the liver.

## 5 Mechanisms through which RIPC activation influences hepatic glucose metabolism and NNCCS

Increased glucose production is a major determinant of fasting hyperglycemia in DM. The activation of the nucleus of the PNS (NST and DMX), suppresses liver gluconeogenesis to regulate blood sugar (BS) homeostasis (19). To further examine the effect of RIPC on hepatic glucose metabolism, we used hindlimb IR-induced male C57BL/6J WT mice to determine whether RIPC activates the PNS, negatively regulates hepatic gluconeogenesis, and enhances glucose uptake (20). IR significantly activated the center of the PNS (NST and DMX), suggesting that it has a role in activating the cholinergic system. This RIPC not only downregulated hepatic gluconeogenic enzymes [glucose-6-phosphatase (G6Pase) and phosphoenolpyruvate carboxykinase (PEPCK)], but also accelerated hepatic glucose uptake through the HIF-1α-GLUT4 pathway. IR increased the expression of GLUT4 in the plasma membrane, but not in the cytosol in the liver. Thus, IR reduced BS levels by approximately 20%. Hepatic branch vagotomy in mice subjected to IR attenuated the decrease in BS levels. As ChAT-tgm, ChAT, HIF-1, and GLUT4 protein levels were also upregulated in the heart, intracerebroventricular administration of a ChAT inhibitor attenuated not only the expression of the gluconeogenic enzyme, but also the expression of cardiac ChAT. This suggests that cardiac ACh synthesis through NNCCS, which is activated by RIPC via PNS, including CNS, influences hepatic glucose metabolism.

## 6 Effect of RIPC and NNCCS on energy metabolism in the diabetic liver

In ChAT-expressing HEK293 cells, NNCCS plays an important role in cellular energy metabolism to enhance glucose metabolism with glucose uptake, reciprocally negatively modulating mitochondria-mediated energy metabolism, sustaining adenosine triphosphate (ATP) levels by suppressing energy consumption, and resists to prolonged serum deprivation (8, 12, 18). Similar results were observed in cardiomyocyte cell lines HL-1 and H9c2 cells, and the hindlimb IR increased ACh and ATP levels *in vivo* murine heart (12). Oikawa et al. suggested that RIPC suppresses PDK4 expression, which is a negative regulator of pyruvate dehydrogenase (PDH) in *in vivo* murine heart (22). The latter regulates the step from glycolysis to the tricarboxylic acid (TCA) cycle and maintains high ATP levels. This suggests that glucose metabolites efficiently enter the TCA cycle by RIPC in the heart. In our study, hindlimb IR significantly attenuated hyperglycemia in murine models of type 1 and type 2 DM (20). The liver is the largest organ which plays an important role in glucose metabolism. Moreover, as mentioned above, RIPC may act on the liver. Therefore, we examined the mechanisms by which hindlimb IR regulates energy metabolism in the liver from STZ-induced T1DMm (35). Serum ketone body levels were significantly decreased in IR-treated T1DMm subjects under feeding conditions. Consequently, IR significantly enhanced the enzyme activity of PDH and aconitase in the T1DMm liver, suggesting it promotes glucose utilization by activating the TCA cycle in the diabetic liver. IR activates AMP-activated protein kinase α, which suppresses ATP consumption and enhances mitochondrial ATP production, and maintains cellular energy reserves (35, 36). Furthermore, the expression of peroxisome proliferator-activated receptor γ coactivator-1 (PGC-1) protein in the IR-treated T1DM liver was significantly lower compared with that in the non-IR-treated T1DM liver. PGC-1α is a fasting-induced transcriptional coactivator that induces gluconeogenesis and fatty acid oxidation, while inhibiting glycolysis (37). Under fasting conditions, PGC-1 binds to Sirt1 to form a protein complex and enhances the transcriptional activity of its target genes (i.e., PEPCK and G6Pase) (38, 39). As mentioned above, IR suppresses hepatic gluconeogenesis by downregulating the expression of G6Pase and PEPCK, while enhancing glucose uptake (20). Low PGC-1 expression may be the result of the IR-mediated improvement of dysfunctional hepatocellular glucose uptake in DM. These findings support our hypothesis that the shift in substrate preference during hepatic energy metabolism from glucose to fatty acid under T1DM conditions is suppressed by RIPC.

In our study, blood non-esterified fatty acid (NEFA) levels were significantly higher in IR-treated T1DMm compared with non-IR-treated T1DMm and non-IR-treated non-T1DMm subjects (35). We speculated that a decrease in fatty acid consumption associated with low PGC-1 expression by IR may contribute to these results. However, the details of the physiological or pathological effects of increased NEFA (e.g., changes in insulin sensitivity, changes in triglycerides and cytokine blood concentrations, and changes in incidence of atherosclerotic lesions) are unknown. NEFA causes insulin resistance, endothelial dysfunction, and activation of proinflammatory pathways in skeletal muscle, liver, and endothelial cells, and is closely associated with atherosclerotic vascular disease (40). At least, our previous studies have not shown that IR affected insulin sensitivity using C57BL/6J WT mice (20). Activation of cardiac ACh synthesis through NNCCS, which is activated by RIPC, may increase ATP production through glucose, rather than fatty acid, in the liver under diabetic conditions, and may not only prevent diabetic hyperglycemia, but also ketosis. However, the longer period studies may be needed to consider the clinicopathological effect of these NEFA increasing by RIPC and/or NNCCS.

## 7 Histological study of the effect of RIPC and NNCCS on hepatic glucose metabolism

GLUT4 is the isoform localized in the skeletal muscle, fat tissues, and heart, rather than the liver (41). GLUT4 exists primarily as an intracellular disposition in the unstimulated state and is acutely redistributed through the plasma membrane in response to insulin and other stimuli for glucose uptake (42, 43); however, the isoform localized in the liver is primarily GLUT2 (44). Using IR-treated C57BL/6J WT mice, we measured the expression of GLUT4 and GLUT2 in each zone of the hepatic lobules (zone 1: periportal area, zone 2: intermediate area, and zone 3: central area) under feeding conditions (45) by immunohistochemistry. In each zone, particularly zone 2, the staining intensity of GLUT4 in the IR-treated liver was significantly higher compared with that in the non-IR-treated liver. In contrast, GLUT2 expression in each hepatic zone was comparable between the IR- and non-IR-treated control liver. The GLUT4 mRNA levels in the IR-treated liver were also significantly increased compared with that in the control group, whereas that of GLUT2 was comparable. In ACh-treated primary cultured hepatocytes, ACh upregulated GLUT4 expression on the membrane periphery, which indicates that GLUT4 is translocated to the plasma membrane. Braeuning et al. found that genes encoding enzymes participating in glycolysis were preferentially expressed in zone 3, where oxygen tension was lower, rather than zone 1, whereas those involved in gluconeogenesis and fatty acid degradation were highly expressed in zone 1 (46). Moreover, compared with non-IR-treated livers, marked glycogen accumulation was observed in IR-treated livers, especially in zone 1 (45). These results suggest that because of maintaining energy production using glucose incorporated into hepatocytes by enhanced GLUT4, glycogen consumption was suppressed, which is consistent with our results demonstrating that energy production was promoted through the acceleration of the TCA cycle and associated with increased glucose preference in the IR-treated liver (35). This continuous energy production with glucose preference, especially in zone 1 and zone 2, may contribute to the reduction of BS levels by RIPC. Thus, activation of NNCCS may contribute to GLUT4 overexpression in the periportal area where fatty acid degradation occurs. Then, NNCCS may lead to suppress ketosis in T1DMm.

## 8 Conclusion

**Figure 1:**
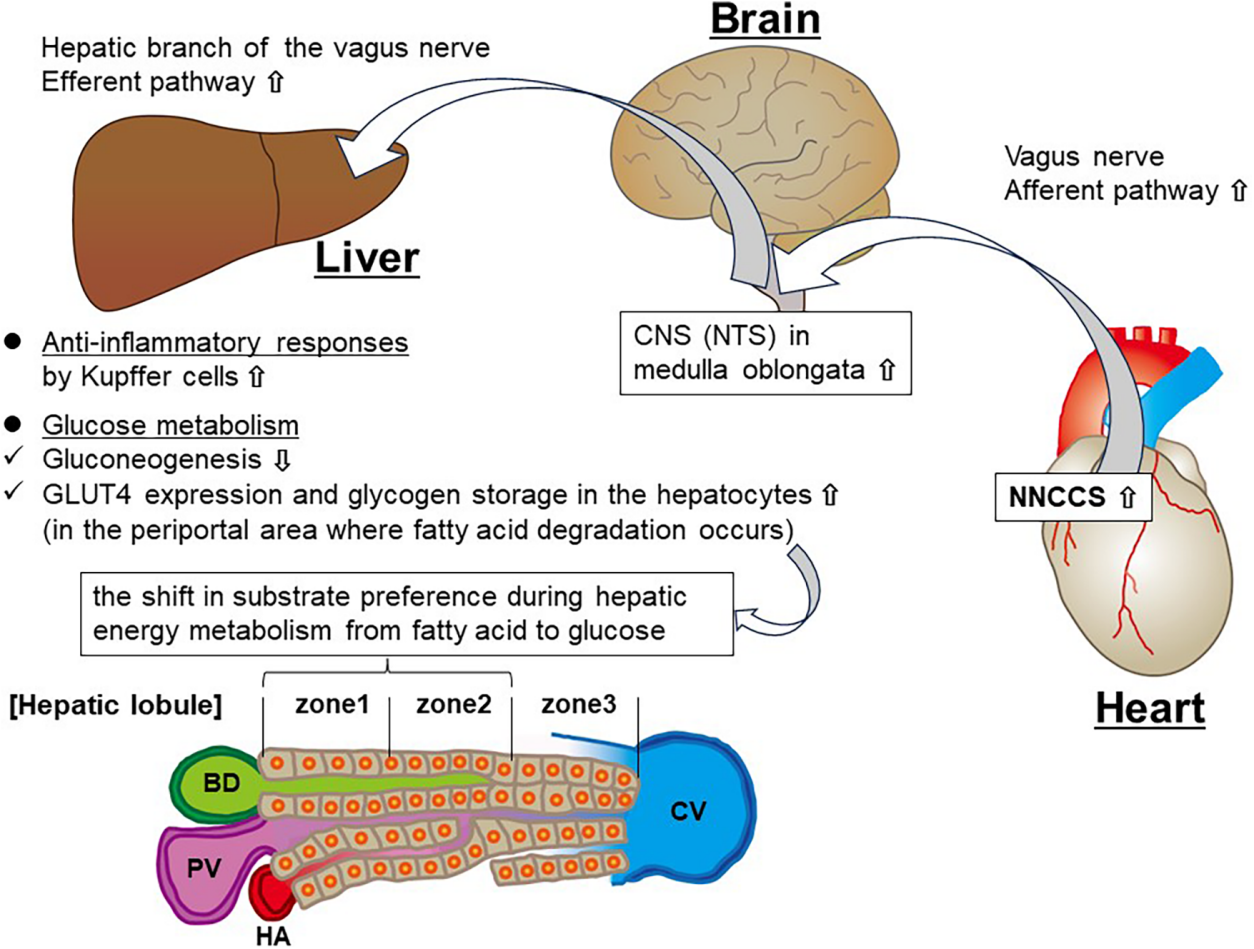
Schema showing the hypothesized link between augmented NNCCS and the liver. PV, portal vein; BD, bile duct; HA, hepatic artery; CV, central vein.

In the heart, NNCCS upregulates the expression of GLUT4 and glucose uptake and participates in energy conservation to sustain ATP levels (11). Hindlimb IR also upregulated the expression and glucose uptake in the liver (20). Moreover, hindlimb IR increases ATP production using glucose rather than fatty acids in T1DMm (35). Both NNCCS and RIPC activate the CNS. Taken together, NNCCS may directly contribute to glucose and energy metabolism, which accelerates the TCA cycle associated with increased glucose preference in the liver via the CNS and vagus nerve. On the flip side of this glucose metabolism, the cholinergic system, including the vagal nerve, plays an anti-inflammatory role in peripheral inflammatory responses (28). The schema of this hypothesized link between augmented NNCCS and other organs is summarized in Figure 1. NNCCS may offer a beneficial therapeutic modality for controlling diabetic hyperglycemia and its complications (e.g., ketoacidosis), including hepatic chronic inflammatory disease (e.g., steatohepatitis). However, there is no direct evidence of NNCCS in human disease. This should be the subject of future laboratory and clinical studies to determine the effect of NNCCS on disorders of glucose metabolism.
